# A Hybrid Scheme for Fine-Grained Search and Access Authorization in Fog Computing Environment

**DOI:** 10.3390/s17061423

**Published:** 2017-06-17

**Authors:** Min Xiao, Jing Zhou, Xuejiao Liu, Mingda Jiang

**Affiliations:** 1 School of Cyber Security and Information Law, Chongqing University of Posts and Telecommunicaitons, Chongqing 400065, China; xiaomin@cqupt.edu.cn; 2 College of Computer Science and Technology, Chongqing University of Posts and Telecommunicaitons, Chongqing 400065, China; s140201109@stu.cqupt.edu.cn (J.Z.); s140231021@stu.cqupt.edu.cn (M.J.); 3 Institute of Service Engineering, Hangzhou Normal University, Hangzhou 311121, China

**Keywords:** searchable encryption, attribute-based encryption, online/offline encryption, mediated encryption, cloud computing, fog computing

## Abstract

In the fog computing environment, the encrypted sensitive data may be transferred to multiple fog nodes on the edge of a network for low latency; thus, fog nodes need to implement a search over encrypted data as a cloud server. Since the fog nodes tend to provide service for IoT applications often running on resource-constrained end devices, it is necessary to design lightweight solutions. At present, there is little research on this issue. In this paper, we propose a fine-grained owner-forced data search and access authorization scheme spanning user-fog-cloud for resource constrained end users. Compared to existing schemes only supporting either index encryption with search ability or data encryption with fine-grained access control ability, the proposed hybrid scheme supports both abilities simultaneously, and index ciphertext and data ciphertext are constructed based on a single ciphertext-policy attribute based encryption (CP-ABE) primitive and share the same key pair, thus the data access efficiency is significantly improved and the cost of key management is greatly reduced. Moreover, in the proposed scheme, the resource constrained end devices are allowed to rapidly assemble ciphertexts online and securely outsource most of decryption task to fog nodes, and mediated encryption mechanism is also adopted to achieve instantaneous user revocation instead of re-encrypting ciphertexts with many copies in many fog nodes. The security and the performance analysis show that our scheme is suitable for a fog computing environment.

## 1. Introduction

Cloud computing enables on-demand network access to the ample computation and storage resources and has been a dominant computing paradigm in recent years. However, Internet of Things (IoTs), an emerging wave of Internet deployments, requires mobility support, geo-distribution, location awareness and low latency and poses a challenge to the centralized cloud computing system [1]. For solving the challenge, a new platform working between end users and cloud data centers, fog computing is proposed by Cisco recently to provide data, compute, storage and application services to end users and extend cloud computing and services to the edge of the network [1,2]. When sensitive data needs to be stored on fog nodes untrusted as public cloud platform, data encryption and search over encrypted data are still preferred approaches for data confidentiality. However, fog computing inserts a middle layer into the infrastructure of cloud computing to form a three-layer computing architecture with resource constrained end users, and data security protection mechanisms in fog computing need to span user-fog-cloud and meet the resource constraints at different levels [3].Thus the approaches in traditional cloud computing may not entirely appropriate for fog computing environment. Currently, works on security or privacy issues in fog computing are on the increase.

There have been many practical cryptographic schemes designed for data confidentiality in cloud computing environment. Attribute-based encryption (ABE) [4,5] allows flexible one-to-many encryption without prior knowledge of who will be accessing the data and attracts the most attention for fine-grained access authorization over outsourced data. In ABE, the identifier of a user is described by some attributes and the authorized attribute sets constitute access policy which can be embedded into key (key-policy ABE, KP-ABE) [4] or ciphertext (ciphertext-policy ABE, CP-ABE) [5]. For CP-ABE, a user with attributes satisfying an access policy can access the data encrypted under the access policy. Therefore, CP-ABE can achieve owner-enforced fine-grained access control on outsourced data [6]. To date, ABE is considered as the best solution for noninteractive access control in cloud computing environment. At present, several works have attempted to apply ABE to fog computing environment for fine-grained access control and they pay attention to security enhancement of ABE for resisting possible new attacks in fog computing [7,8,9] or alleviation of burdens on resource constrained end users [10].

The searchable encryption (SE) [11] is another cryptography mechanism suitable for cloud computing. By SE, the cloud server can perform secure search over encrypted data on the behalf of users. The basic principle of SE is as follows: a data owner generates searchable encrypted indexes for his outsourced files and then a data user can generate some search trapdoors based on the granted key and keywords that interested him, meanwhile, it is required that nothing should be leaked from the trapdoors, indexes or pattern of search queries [12]. The typical application scenarios of SE can be classified into two categories, namely single-user search [13] and multi-user search setting [14,15,16]. ABE is also used to realize fine-grained owner-enforced search authorization in multi-user setting by the following way [16]: the data owners encrypt index under owner-enforced access policies, then data users search the interesting keywords over the encrypted datasets by sending trapdoors generated with their attribute secret keys, unless the attributes of a user satisfy the owner-enforced access policies, the user cannot obtain valid search results. As far as we know, there is no SE scheme designed for fog computing environment.

There have been some attempts to combine data encryption and searchable encryption into a hybrid solution for protection of outsourced data. After Boneh et al. [17] presented the first public key encryption with keyword search (PEKS) in 2004, Baek et al. [18] pointed out that a PEKS scheme is only meaningful when coupled with a public key encryption (PKE) and first considered to combine PKE and PEKS in a secure manner. They also concluded that simply appending a PEKS ciphertext that encrypts a keyword to a PKE ciphertext that encrypts a file is vulnerable to the swapping attack (which can alter the relationship between PEKS ciphertext and PKE ciphertext). Therefore, they suggested that some mechanism should be provided to bind two ciphertexts together and detect any alteration of two ciphertext relationship en route. To date, there is only a small amount of research on this issue and most of which consider single-user search setting. To the best of our knowledge, the scheme in [19] is the only hybrid scheme supporting multi-user search. However, the scheme is constructed based on KP-ABE and CP-ABE is considered to be more appropriate for data protection in cloud storage systems because it gives data owners the ability to select an access policy and to encrypt data under this policy. Obviously, compared to two separate schemes, the hybrid scheme can significantly improve access efficiency and greatly reduce the cost of key management when two ciphertext components share the same key. Therefore, the hybrid scheme is more suitable for IoT applications often running on resource constrained end devices.

By performance test in well-known IoT platforms, Ambrosin et al. [20] have demonstrated that adopting ABE in the IoT is indeed feasible. In this paper, we focus on the design of a hybrid scheme based on CP-ABE in multi-user access setting and fog computing environment. The work needs to address the following challenges.

- On the promise of ensuring the security of cryptography scheme, the index encryption and data encryption components are integrated under the same access policy and key pair to achieve higher efficiency at a lower cost.
- In ABE scheme, the computational costs of the encryption and decryption scale with the complexity of the access policy or number of attributes and are primarily incurred by complex exponentiations and pairing operations. Although Ambrosin et al. [20] declared the feasibility of ABE for IoT devices, they also advised the migration of complex arithmetic operations to more powerful parties in order to enhance energy efficiency and total execution time. Therefore, how to alleviate the burden on resource-constrained end users and solve the possible performance bottleneck caused by resource limitation is still a problem that needs to be considered in scheme design.
- In the fog computing environment, many copies of encrypted data can be generated and distributed to many fog nodes, but the ciphertexts of the basic ABE scheme need to be re-encrypted when a user revocation occurs, and consequently, directly applying the basic ABE scheme to fog computing environment will be inefficient due to huge revocation cost.

Fortunately, there have been some measures to solve the above challenges. ABE with outsourced decryption [21,22] and online/offline ABE [23] are designed to address performance bottleneck problem from decryption and encryption operations on the devices with limited resources, respectively. In addition, mediated CP-ABE [24] enables instantaneous user revocation. We incorporate the above techniques to design a hybrid search and access authorization scheme for encrypted data in fog computing environment. In the proposed scheme, the search trapdoor is generated with the help of a mediator (fog node), and thus instantaneous user revocation is achieved as long as the fog node stops cooperating with the revoked user. In particular, the search trapdoor is also used as a decryption token by which fog node can pre-decrypt data ciphertext into a simple ElGamal-style ciphertext to greatly alleviate burdens on end user. To the best of our knowledge, it is the first hybrid scheme achieving fine-grained owner-enforced search and access authorization simultaneously over encrypted data in multi-user setting. 

### 1.1. Related Works

#### 1.1.1. Fine-grained Access Control Based on ABE

ABE was first constructed from IBE by Sahai et al. [25] and then Goyal et al. [4] classified ABE into two main types: KP-ABE and CP-ABE. The ABE technique plays an important role in cloud security and more attention is paid to CP-ABE for performing owner-enforced access policy over outsourced data. The first CP-ABE scheme [5] supports monotonic access structure and is proved secure under the generic group heuristic. Afterwards, Cheung et al. [26] proposed a selectively secure CP-ABE scheme with simple access structure (consisting of a series of AND gates) under the standard model. When designing practical ABE schemes, the attribute/user revocation is the main challenge and concern. A general solution to address the problem is the timed rekeying mechanism [5,27], in which an expiration time is appended to each of the attributes and key update is implemented periodically. The proxy re-encryption mechanism [28,29] can delegate most of update tasks to semi-trusted proxy servers and thus is more suitable for cloud computing environment. In the above revocation mechanisms, the update costs are linearly related to the number of users and ciphertexts in system and instantaneous user revocation is difficult. Luan I. et al. [24] proposed a mediated CP-ABE (mCP-ABE) scheme, in which the user secret key is divided into two shares, one share for the semi-trusted mediator and the other for the user. When decrypting a ciphertext, the user must contact the mediator to receive a decryption token. When a user/attribute revocation occurs, the mediator refuses to issue the decryption token for the revoked user/attribute, thus instantaneous revocation is implemented. ABE with outsourced decryption (OD-ABE) [21,22] and online/offline ABE (OO-ABE) [23] were proposed for resource-constrained end devices and are perfectly suitable for mobile cloud and IoT environments. OD-ABE is designed to move most of the decryption cost on ABE ciphertexts to a semi-trusted proxy. In OO-ABE, most of encryption task can be implemented during device’s idle time and then the device can rapidly assemble ABE ciphertexts online.

At present, several ABE schemes have been advised for fine-grained access control in fog computing environment. Some researchers consider that the sensitive data stored in fog computing might be encountered more sophisticated attacks and focus on design of ABE scheme with higher security. Zuo, C. [7] proposed a CCA-secure ABE scheme with outsourced decryption and Yinhao et al. [8] presented a novel CP-ABE mechanism to address the possible key-delegation abuse issue in fog computing. Yu, Z. et al. [9] construct functional encryption schemes adaptively secure in continual memory leakage model (CML) to not only provide privacy preserving and fine-grained access control in fog computing but also resist side channel attacks. Considering the resource restraints of end devices in fog computing, Zhang, P. et al. [10] presented a CP-ABE scheme which outsources the heavy computation operations of encryption and decryption to fog nodes. However, the scheme requires cloud server and fog nodes to be trusted and does not achieve instantaneous user revocation.

#### 1.1.2. Searchable Encryption

The privacy-preserving search over various types of data has been widely studied. A plenty of searchable encryption (SE) schemes are proposed by researchers to implement a designated single user search. Song et al. [11] presented the first practical searchable encryption scheme, which realizes full text search without loss of confidentiality. For speeding up the search process, secure index per document mechanism [13] was proposed to reduce the search time to the number of documents. In 2004, Boneh et al. [17] constructed the first public key encryption with keyword search (PEKS) scheme, which allows multiple users to generate searchable ciphertexts based on public key and a private key holder to search over the ciphertexts. There are many variants of PEKS. In particular, Baek et al. [18] and Zhang et al. [30] paid attention to the problem of combining PKE and PEKS in a secure manner. However, their proposed schemes are still based on two independent primitives with different key pairs and a tagging mechanism binds PKE and PEKS together to resist the swapping attack. Afterwards, Chen et al. [31] constructed a hybrid scheme based on one IBE primitive with the same key pair for both PKE and PEKS components, thus the number of keys are greatly reduced. However, the scheme only supports single-user search.

In a multi-user search setting, the data owner creates searchable content and an owner-defined group is allowed to generate trapdoors and access protected data. Therefore, the key distribution and user revocation are two important requirements and challenges. Some classical technologies, such as broadcast encryption [14] and secret sharing [32], are used to distribute the secret values to a group of users but user revocation cost grows linearly with the number of users and ciphertexts in the system. By introducing a trusted third party (TTP) [33] or semi-trusted third party (sTTP) [15] to transform the searchable ciphertexts for different users in a group or cooperate with legitimate users for the generation of valid trapdoors, the instantaneous user revocation can be achieved as long as TTP/sTTP stops service for the revoked user. However, only relying on a third party, the user authorization is coarse-grained. Sun et al. [16,34] applied ABE technique in [26] to construct the first attribute-based keyword search scheme (ABKS) for fine-grained (file-level) owner-enforced search authorization in multi-user setting. Moreover, the proxy re-encryption and lazy re-encryption techniques are adopted to improve the efficiency of user revocation, but the instantaneous user revocation can not be supported. In [19], a KP-ABE based hybrid scheme supporting keyword private search and data encryption/decryption simultaneously is proposed, but it can not implement owner-enforced authorization and instantaneous user revocation. To date, there has been no hybrid scheme designed for fog computing environment. In the following, we list the differences of above works in Table 1.

### 1.2. Contributions

The main contributions of this paper are listed below:

- For the new cloud computing architecture with fog computing layer, we design a hybrid authorization model spanning user-fog-cloud, by which the authorized users can securely delegate search task over encrypted data to cloud server/fog nodes and decrypt the search results with the help of fog nodes.
- To improve access efficiency, reduce key management cost and resist swapping attack, the data ciphertext and index ciphertext are constructed based on the same access policy and key pair.
- To meet resource constraints of end IoT devices, we incorporate decryption outsourcing technique of ABE to outsource most of decryption computation task to fog node, and meanwhile, we also adopt online/offline ABE technique to calculate in advance most of ciphertext components during device’s idle time and then the device can rapidly assemble ABE ciphertexts online. Thus, energy efficiency and total execution time on end devices are enhanced and the possible performance bottleneck is avoided.
- Considering that many copies of encrypted data are distributed to many fog nodes, we incorporate mediated ABE technique to achieve instantaneous user revocation and avoid huge revocation cost incurred by re-encryption of ciphertexts.
- We prove the proposed scheme is selectively secure against chosen keyword attack (CKA) and chosen plaintext attack (CPA) under standard model. Performance evaluation shows that the proposed scheme can effectively protect data confidentiality and achieve secure data sharing in fog environment.

The remaining paper is organized as follows. In Section 2, the system overview is described. Our scheme are presented in Section 3. Security and performance analysis are given in Section 4. Finally, we conclude the paper in Section 5.

## 2. System Overview

### 2.1. System Model

**Figure 1:**
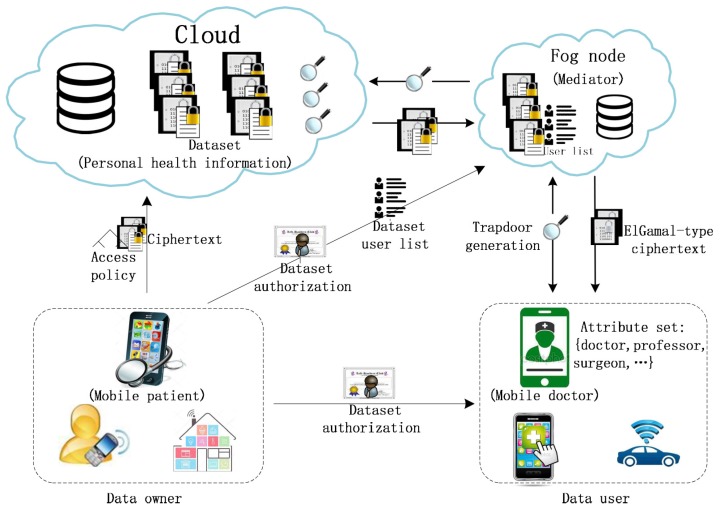
System Model.

The system includes several entities, data owner, cloud server, fog node and many users, as shown in Figure 1.

**Data owner** determines the access policy of each data file, encrypts data files and generates secure indexes under the designated policies by our proposed scheme before he uploads his data into cloud server. For accelerating search process, the data owner can divide his data into multiple datasets, and for each dataset, the data owner performs a coarse-grained authorization by authorizing data users and designated fog nodes and creating a user list (UL). When a user revocation occurs, the data owner adds the revoked user to user revocation list (URL).

**Cloud server** stores encrypted data for owners and honestly does search over the encrypted data on behalf of data user and returns the results to the designated fog node.

**Fog node** acts as a mediator between data user and cloud server for trapdoor generation, caches some datasets frequently accessed for rapid response to local users and undertakes most of data decryption tasks for end users. When receiving a search request from a data user in UL, the fog node cooperates with the data user to generate search trapdoor and first performs a local search over its cache, if there is no desired result, the fog node submits the trapdoor to cloud server. When receiving a search result from search engine, using corresponding search trapdoor, the fog node can pre-decrypt the retrieved ciphertexts into simple ElGamal-type ciphertexts and sends them to the data user. The fog nodes will refuse to cooperate with the revoked users, thus the revoked users cannot submit valid trapdoors to cloud server and are revoked immediately.

**Data user** interacts with the chosen fog node to generate search trapdoors for access to some encrypted data stored in cloud. When receiving the search results from a fog node, he can rapidly decrypt the ElGamal-type ciphertexts into data plaintexts.

In addition, a fully trusted **attribute authority (AA)** of ABE scheme (not shown in Figure 1) is needed to verify the entities in the system and distribute public parameters and private keys. Particularly, each data user’s attribute private key is divided into two parts by AA: the one is for the data user and the other is for the designated fog nodes.

### 2.2. An Application Scenario

Figure 1 also presents an application scenario of our scheme in mobile healthcare environment, where a patient is promised the full control and management of her person health information (PHI) from different sources (such as wearable health sensors, hospital etc.), and the PHI files must be available anywhere and anytime to mobile pervasive healthcare services including mobile telemedicine, patient monitoring, location-based medical services, emergency response and management, and so on. In order to made the storage, retrieval and sharing of PHI more efficient, the patient outsources his PHI files to cloud. Since PHI is highly sensitive data that is related to the patient interests, the PHI files should be encrypted before outsourcing. Using our proposed scheme, the PHI owner herself decides how to encrypt her PHI files and to allow which set of users to obtain search and access authorization of each file by selecting appropriate access policy, moreover, the fog nodes widely distributed deployment promote timely and pervasive access to PHI files by search over the encrypted PHI datasets in cloud or cached in fog nodes. In particular, even if the data owner or data user is resource-constrained, the efficiency of the storage, retrieval and sharing of PHI can also be ensured very well.

### 2.3. Threat Model and Security Goals

We consider that cloud server is semi-trusted (i.e., honest-but-curious). That is to say, cloud server would follow the designated protocol honestly to store data for owners and perform search over encrypted data for end users, but it is curious about the sensitive information, such as the keywords to be searched, relationship between trapdoors and sensitive data in its storage system. The fog nodes are also semi-trusted, which store shares of user secret keys without leakage and perform tasks required by the protocol honestly. However, the fog nodes may be curious about the searched keywords, relationship between trapdoors and sensitive data that they cache. The users are malicious and try to collude with each other to search and decrypt data beyond their access rights, but these users are not allowed to collude with the fog nodes.

Under the above security model, the security goals of the proposed scheme are described as follows:

- Keyword privacy: The proposed hybrid scheme can achieve selectively secure against chosen-keyword attack (CKA). We will prove it under standard model in Appendix A.
- Data confidentiality: The proposed hybrid scheme can achieve selectively secure against chosen-plaintext attack (CPA). We will prove it under standard model in Appendix A.
- Trapdoor unlinkability: This security property makes the cloud server or fog nodes unable to visually distinguish two or more trapdoors even containing the same keyword.
- Swapping attack resistance: This security property requires that the alteration of relationship between index ciphertext and data ciphertex can be detected immediately.

Note that, as pointed in [16,34], the public key encryption based search scheme can not protect the predicate privacy because the attacker may launch dictionary attack by using public key to generate arbitrary number of indexes with keyword of his choice, and then search these indexes with a particular trapdoor to deduce the underlying keyword in the trapdoor. Therefore, our scheme does not consider protection of predicate privacy.

## 3. Proposed Hybrid Scheme

Our scheme considers fine-grained owner-enforced search and access authorization over outsourced sensitive data in a three-layer architecture spanning user-fog-cloud. Compared to the authorization scheme in classical cloud service architecture, the proposed scheme applies an additional middle layer, fog nodes, to lower access delay, alleviate burdens on end users and reduce user revocation cost. In the following, we present three main phases of our scheme, namely system initialization, sensitive data outsourcing storage and search and access of outsourced sensitive data.

### 3.1. System Initialization

In this phase, system public parameters and master key are set and the entities enroll themself into this system.

#### 3.1.1. System Setup

The attribute authority (AA) in the system first chooses a security parameter λ according to the required security level, then defines a bilinear group *G* of prime order *p* with a generator *g* and a bilinear map $e:G\times G\to G_{T}$, which has the properties of bilinearity, computability and non-degeneracy [25]. AA also chooses a collision-resistant hash function $H:{\left\{0,1\right\}}^{*}\to Z_{P}$ and, randomly picks a secret value $y\in Z_{P}$ and computes $Y=e{\left(g,g\right)}^{y}$.

Assume that the number of attributes in the system is *n* and the universal attribute set is N={1,⋯,n}. AA chooses a series of random elements $\left\{t_{1},\ldots ,t_{3n}\right\}$ from $Z_{P}$ and for each k∈{1,⋯,3n}, it calculates $A_{k}=g^{t_{k}}$. For i∈N, $A_{i}$ are referred to as *positive* attributes, $A_{i+n}$ are for *negative* attributes and $A_{i+2n}$ are for *don’t care* attributes [26].

Finally, AA keeps $MSK=(y,t_{1},\cdots ,t_{3n})$ as secret and publishes public parameters $PP=(g,p,e,H,Y,A_{1},\cdot \cdot ,A_{3n})$ to the system.

#### 3.1.2. Mediator Register

When a fog node joins the system to act as a mediator, the register process includes two steps. First, the fog node requests AA for key distribution. AA chooses a random $\gamma \in Z_{p}$ and computes $PP_{med}=Y^{\gamma }$, where $PP_{med}$ is published as mediator’s public key while γ is kept as secret value by the fog node. Next the data owner authorizes the fog node as a mediator of a dataset by distributing a dataset-mediator public key $D_{med}^{set}=PP_{med}^{-s}=Y^{-\gamma s}$ and per-dataset user list (UL) to the fog node, where *s* is a secret value related to the dataset and is shared by each data file in the dataset.

#### 3.1.3. User Register

Any user that wants to join the system, has to submit the certification of his identity UID and attributes set *S* to AA to ask for secret key. AA first selects a secret value $u\in Z_{p}$ randomly and computes user identity public key $PP_{UID}=Y^{u}$. Then, AA generates two shares of the user secret key. For each i∈{1,⋯,n}, AA chooses $r_{i},x_{i}\in Z_{p}$ randomly and computes

$$K_{i,1}=\left\{\begin{array}{cc} g^{\left(r_{i}-x_{i}\right)/t_{i}} & i\in S\\ g^{\left(r_{i}-x_{i}\right)/t_{i+n}} & i\notin S \end{array}\right.,\;K_{i,2}=\left\{\begin{array}{cc} g^{x_{i}/t_{i}} & i\in S\\ g^{x_{i}/t_{i+n}} & i\notin S \end{array}\right.$$

Moreover, it has $F_{i,1}=g^{\left(r_{i}-x_{i}\right)/t_{i+2n}}$ and $F_{i,2}=g^{x_{i}/t_{i+2n}}$ for all i∈{1,⋯,n}. AA also computes $K_{1}=g^{y-r}$, where $r=\sum r_{i}$. Finally, the AA sends $\left(K_{1},{\left\{K_{i,1},F_{i,1}\right\}}_{i\in N}\right)$ to the authorized mediators while the secret value *u* and $\left({\left\{K_{i,2},F_{i,2}\right\}}_{i\in N}\right)$ are sent to the user.

In addition, if a user never performs search over a dataset before, he must request the data owner for authorized access to the dataset. The data owner computes a dataset-user public key $D_{UID}^{set}=PP_{UID}^{-s}=Y^{-us}$, then he asks the mediator to add the tuple $\left(UID,\;D_{UID}^{set}\right)$ to the per-dataset UL.

### 3.2. Sensitive Data Outsourcing Storage

The data owner enforces a two-level authorization model, namely coarse-grained authorization at dataset level using per-dataset UL and fine-grained authorization at file level using per-file access policy. Before outsourcing sensitive data to cloud server, the data owner generates secure index for each data file and encrypts the data under the designated access policy. Considering the resource limitation of end IoT devices, our scheme exploits the online/offline ABE technique in [23] to split the encryption computation tasks into two steps, namely offline computation and online computation. The offline computation does the vast majority of the work to encrypt index and data file before knowing the data and access policy, and the online computation can rapidly assemble a secure index and data ciphertext. In particular, the offline computation can be performed while the device is idle or plugged into a power source.

#### 3.2.1. Offline Computation

In this step, an arbitrary number of intermediate ciphertexts are created by data owner during his idle time to obtain a resource pool. The intermediate ciphertext includes most of ciphertext components and can be used to rapidly assemble a complete ciphertext online.

According to [23], the intermediate ciphertext includes two types of modules, namely main module and contribute module, which can be independently created.

- Main Module Generation: The data owner picks a random number $\eta \in Z_{p}$ and computes $\Phi_{0}=Y^{\eta },\bar{\Phi }=g^{\eta }$. The tuple $\left(\eta ,\Phi_{0},\bar{\Phi }\right)$ is a main module. The data owner can generate an arbitrary number of main modules.
- Attribute Module Generation: For each k∈{1,…,3n}, the data owner selects a random $\varphi_{k}\in Z_{p}$ and computes $\Phi_{k}=A_{k}^{\varphi_{k}}$ and each tuple $\left\{\varphi_{k},\Phi_{k}\right\}$ is called as an attribute module. The data owner can generate an arbitrary number of attribute modules for each *k*.

The main modules and attribute modules constitute a resource pool Σ.

#### 3.2.2. Online Computation

For a data file *m*, the data owner specifies an exact access policy $GT=\wedge_{i\in I}\underline{i}$ and a keyword *w*, where literal $\underline{i}$ is either positive *i* or negative ¬i. Then, the data owner generates secure index containing the keyword *w* and data ciphertext under the access policy GT.

• Secure Index Generation

In order to implement a two-level authorization structure, for a dataset, the data owner randomly selects a main module and for a data file in the dataset, *n* attribute modules are chosen randomly from the pool Σ. The rule selecting attribute modules is as follows: if $i\in I,\underline{i}=i$, an attribute module $\left\{\varphi_{i},\Phi_{i}\right\}$ is selected randomly; if $i\in I,\underline{i}=\neg i$, an attribute module $\left\{\varphi_{i+n},\Phi_{i+n}\right\}$ is selected randomly and if i∈N∖I, an attribute module $\left\{\varphi_{i+2n},\Phi_{i+2n}\right\}$ is selected randomly. Consequently, a main module and *n* attribute modules constitute the intermediate index ciphertext of a data file, denoted as $II=(s,D_{0},\bar{D},{\left\{v_{k},D_{k}\right\}}_{k\in \{i,i+n,i+2n\},i\in N})$. Note that, the intermediate index ciphertexts of all data files in the same dataset share the same main module, which is used to implement coarse-grained authorization at dataset level, while each data file has different attribute modules, which will be associated to access policy of a data file and used to implement fine-grained authorization at file level.

Then, the complete secure index $CT_{w}=\left(D_{0},\bar{D},{\left\{D_{i,1},D_{i,2}\right\}}_{i\in N}\right)$ can be created by the following calculation.

$$D_{i,1}=\left\{\begin{array}{ccc} D_{i} & =A_{i}^{v_{i}} & i\in I,\underline{i}=i\\ D_{i+n} & =A_{i+n}^{v_{i+n}} & i\in I,\underline{i}=\neg i\\ D_{i+2n} & =A_{i+2n}^{v_{i+2n}} & i\in N\setminus I \end{array}\right.\;D_{i,2}=\left\{\begin{array}{cc} s-v_{i} & i\in I,\underline{i}=i\\ s-v_{i+n} & i\in I,\underline{i}=\neg i\\ s-v_{i+2n} & i\in N\setminus I \end{array}\right.$$

Specially, for some $i^{\prime }$, $D_{i^{\prime },1}=D_{i^{\prime }}^{1/H(w)},D_{i^{\prime },2}=\left(s-v_{i^{\prime }}\right)/H\left(w\right)$, where H(w) is a hash value of the keyword *w*. Without loss of generality, the attribute $i^{\prime }$ can be supposed to be positive and this fixed position can be seen as part of public parameter.

• Data Ciphertext Generation

For a data file *m* containing keyword *w*, the data owner randomly selects a main module from the pool Σ. According to the same rule as in secure index generation, *n* attribute modules are also selected randomly. The intermediate data ciphertext is denotes as $IC=(s_{m},C_{0},\bar{C},{\left\{h_{k},C_{k}\right\}}_{k\in \{i,i+n,i+2n\},i\in N})$. Then, the complete data ciphertext $CT_{m}=\left(C_{m},\bar{C},{\left\{C_{i,1},C_{i,2}\right\}}_{i\in N}\right)$ can be created by the following calculation.

$$C_{m}=mC_{0},\;C_{i,1}=\left\{\begin{array}{ccc} C_{i} & =A_{i}^{h_{i}} & i\in I,\underline{i}=i\\ C_{i+n} & =A_{i+n}^{h_{i+n}} & i\in I,\underline{i}=\neg i\\ C_{i+2n} & =A_{i+2n}^{h_{i+2n}} & i\in N\setminus I \end{array}\right.,\;C_{i,2}=\left\{\begin{array}{cc} s_{m}-h_{i} & i\in I,\underline{i}=i\\ s_{m}-h_{i+n} & i\in I,\underline{i}=\neg i\\ s_{m}-h_{i+2n} & i\in N\setminus I \end{array}\right.$$

Specially, for some $i^{\prime }$, $C_{i^{\prime },1}=C_{i^{\prime }}^{1/H(w)},C_{i^{\prime },2}=\left(s-h_{i^{\prime }}\right)/H\left(w\right)$.

Note that, in order to ensure the safety of our scheme, except that the main modules of intermediate index ciphertexts in the same dataset keep the same and can be repeatedly selected from the pool Σ, the other modules in intermediate index and data ciphertexts can only be consumed once and the main modules of intermediate index ciphertexts for different datasets also should be different.

Subsequently, the data owner uploads the $CT=(GT,CT_{w},CT_{m})$ to the cloud server.

### 3.3. Search and Access of Outsourced Sensitive Data

When a data user wants to access data files containing keyword *w* in a dataset, he interacts with some fog node (which may be subscribed by the data user or is the closest to the data user) directly. The fog node looks up URL to confirm whether the user has been revoked and cooperates the non-revoked user to generate search trapdoor if the user is in UL of the dataset, then performs a local search or forwards the search trapdoor to cloud server. Only authorized users with attributes satisfying the access policy of a data file with keyword *w* can obtain a matching result. All valid search results are returned to the fog node, which will pre-decrypt the returned ciphertexts to (constant size) ElGamal-type ciphertexts. The data user only performs one exponentiation and one multiplication operation on each ciphertext to obtain data plaintext.

#### 3.3.1. Trapdoor Generation

When a registered user UID with identity public and secret key pair $\left(Y^{u},u\right)$, dataset-user public key $D_{UID}^{set}$ and attribute set *S* wants to search some data files with keyword $w^{\prime }$ in a dataset with secret value *s*, he generates a search trapdoor with the help of a chosen fog node with identity public and secret key pair $\left(Y^{\gamma },\gamma \right)$ and dataset-mediator public key $D_{med}^{set}$. Note that, in our scheme, neither the fog node nor the user can build a valid trapdoor only by himself. The trapdoor generation process follows the following procedure.

**Step 1**

The user first asks the fog node for search data over a designated dataset, then the fog node checks whether the user is in the UL of the designated dataset and will respond as follows if the user is recorded.

The fog node chooses $\beta \in Z_{p}$ randomly and for all i∈N, he computes

$$Q_{1}=K_{1}^{\beta },Q_{i,1}=K_{i,1}^{\beta },QF_{i,1}=F_{i,1}^{\beta },$$

where $\left(K_{1},{\left\{K_{i,1},F_{i,1}\right\}}_{i\in N}\right)$ is the mediator-side share of the user secret key. Then $T_{med}=\left(Q_{1},{\left\{Q_{i,1},QF_{i,1}\right\}}_{i\in N}\right)$ is returned to the user.

**Step 2**

After receiving the $T_{med}$ from the fog node, the user chooses a random $\alpha \in Z_{p}$ and computes:

$Q_{0}=u+\alpha$, $T_{1}=Q_{1}^{\alpha }$, ${\left\{T_{i,1}=Q_{i,1}^{\alpha },TF_{i,1}=QF_{i,1}^{\alpha },Q_{i,2}=K_{i,2}^{\alpha },QF_{i,2}=F_{i,2}^{\alpha }\right\}}_{i\in N}$, and for $i^{\prime }\in N$, $T_{i^{\prime },1}=Q_{i^{\prime },1}^{\alpha \cdot H(w^{\prime })},TF_{i^{\prime },1}=QF_{i^{\prime },1}^{\alpha \cdot H(w^{\prime })},Q_{i^{\prime },2}=K_{i^{\prime },2}^{\alpha \cdot H(w^{\prime })},QF_{i^{\prime },2}=F_{i^{\prime },2}^{\alpha \cdot H(w^{\prime })}$, where *u* and ${\left\{K_{i,2},F_{i,2}\right\}}_{i\in N}$ are the user-side share of the user secret key.

Then the user sends $T_{u}=(Q_{0},T_{1},\{T_{i,1},TF_{i,1},Q_{i,2},$ $QF_{i,2}{\}}_{i\in N})$ to the fog node.

**Step 3**

After receiving $T_{u}$ from the user, the fog node computes $T_{0}=\beta \left(u+\alpha \right)+\gamma$ and $T_{i,2}=Q_{i,2}^{\beta },TF_{i,2}=QF_{i,2}^{\beta }$, for i∈N. In addition, the fog node also computes $T_{UID}^{set}={\left(D_{UID}^{set}\right)}^{\beta }$. Finally, it delivers the valid trapdoor $T=(T_{0},T_{1},T_{UID}^{set},D_{med}^{set},{\left\{T_{i,1},T_{i,2},TF_{i,1},TF_{i,2}\right\}}_{i\in N})$ to the search engine in cloud server or local storage system.

#### 3.3.2. Search over Ciphertext

When receiving a trapdoor *T*, the search process over ciphertext $CT=(GT=\wedge_{i\in I}\underline{i},\;\;CT_{w}=\left(D_{0},\bar{D},{\left\{D_{i,1},D_{i,2}\right\}}_{i\in N}\right),\;\;CT_{m}=\left(C_{m},\bar{C},{\left\{C_{i,1},C_{i,2}\right\}}_{i\in N}\right))$ is implemented as follows:

**Step 1**

The search engine transforms the stored ciphertext into $CT=(GT=\wedge_{i\in I}\underline{i},\;\;CT_{w}=\left(D_{0},\bar{D},{\left\{D_{i}^{*}\right\}}_{i\in N}\right),\;\;CT_{m}=\left(C_{m},\bar{C},{\left\{C_{i}^{*}\right\}}_{i\in N}\right))$ by the following calculations:

For all i∈N, $D_{i}^{*}=D_{i,1}\cdot A_{i}^{D_{i,2}}=A_{i}^{v_{i}}\cdot A_{i}^{s-v_{i}}=A_{i}^{s}$ and $C_{i}^{*}=C_{i,1}\cdot A_{i}^{C_{i,2}}=A_{i}^{h_{i}}\cdot A_{i}^{s_{m}-h_{i}}=A_{i}^{s_{m}}$

**Step 2**

The search engine computes:

For each attribute i∈I,

if $\underline{i}=i$ and i∈S,
  $$\begin{array}{c} e\left(D_{i}^{*},T_{i,1}\right)=e\left(g^{t_{i}s},g^{\left(r_{i}-x_{i}\right)\alpha \beta /t_{i}}\right)=e{\left(g,g\right)}^{s\alpha \beta (r_{i}-x_{i})}\\ e\left(D_{i}^{*},T_{i,2}\right)=e\left(g^{t_{i}s},g^{x_{i}\alpha \beta /t_{i}}\right)=e{\left(g,g\right)}^{s\alpha \beta x_{i}}, \end{array}$$
If $\underline{i}=\neg i$ and i∉S,
  $$\begin{array}{c} e\left(D_{i}^{*},T_{i,1}\right)=e\left(g^{t_{i+n}s},g^{\left(r_{i}-x_{i}\right)\alpha \beta /t_{i+n}}\right)=e{\left(g,g\right)}^{s\alpha \beta (r_{i}-x_{i})}\\ e\left(D_{i}^{*},T_{i,2}\right)=e\left(g^{t_{i+n}s},g^{x_{i}\alpha \beta /t_{i+n}}\right)=e{\left(g,g\right)}^{s\alpha \beta x_{i}}. \end{array}$$

For i∉I,

$$\begin{array}{c} e\left(D_{i}^{*},TF_{i,1}\right)=e\left(g^{t_{i+2n}s},g^{\left(r_{i}-x_{i}\right)\alpha \beta /t_{i+2n}}\right)=e{\left(g,g\right)}^{s\alpha \beta (r_{i}-x_{i})}\\ e\left(D_{i}^{*},TF_{i,2}\right)=e\left(g^{t_{i+2n}s},g^{x_{i}\alpha \beta /t_{i+2n}}\right)=e{\left(g,g\right)}^{s\alpha \beta x_{i}}. \end{array}$$

For some $i^{\prime }\in N$, if the keyword $w^{\prime }$ in trapdoor *T* is the same as the keyword *w* in ciphertext CT, the following result can be obtained:

$$\begin{array}{c} e\left(D_{i^{\prime }}^{*},T_{i^{\prime },1}\right)=e\left(D_{i^{\prime }}^{*},TF_{i^{\prime },1}\right)=e{\left(g,g\right)}^{s\alpha \beta (r_{i}-x_{i})}\\ e\left(D_{i^{\prime }}^{*},T_{i^{\prime },2}\right)=e\left(D_{i^{\prime }}^{*},TF_{i^{\prime },2}\right)=e{\left(g,g\right)}^{s\alpha \beta x_{i}}. \end{array}$$

**Step 3**

$CT_{m}$ will be returned to the fog node if the following equation holds, namely, attributes in trapdoor *T* satisfy the access policy embedded in CT and the keyword matching succeeds.

$$e\left(\bar{D},T_{1}\right)\prod_{i\in N}e\left(D_{i}^{*},T_{i,1}^{*}\right)e\left(D_{i}^{*},T_{i,2}^{*}\right)=D_{0}^{T_{0}}\cdot T_{UID}^{set}\cdot D_{med}^{set}$$

where for b∈{1,2},

$$T_{i,b}^{*}=\left\{\begin{array}{cc} T_{i,b} & i\in I\\ TF_{i,b} & other \end{array}\right.$$

**Correctness.**

$$\begin{array}{ccc} e\left(\bar{D},T_{1}\right)\prod_{i\in N}e\left(D_{i}^{*},T_{i,1}^{*}\right)e\left(D_{i}^{*},T_{i,2}^{*}\right) & = & e\left(g^{s},g^{(y-r)\alpha \beta }\right)\prod_{i\in N}e{\left(g,g\right)}^{s\alpha \beta r_{i}}\\ & = & e{\left(g,g\right)}^{sy\alpha \beta }\\ & = & e{\left(g,g\right)}^{sy(\alpha \beta +u\beta +\gamma -u\beta -\gamma )}\\ & = & D_{0}^{T_{0}}\cdot T_{UID}^{set}\cdot D_{med}^{set}. \end{array}$$

#### 3.3.3. Data Decryption

In our scheme, the trapdoor *T* is also a decryption token by which the fog node can pre-decrypt the returned data ciphertext $CT_{m}$ to an ElGamal-style ciphertext and the end user only performs one exponentiation and one multiplication operation to get the plaintext *m*.

**Step 1: Fog node predecryption**

According to trapdoor $T=(T_{0},T_{1},T_{UID}^{set},D_{med}^{set},{\left\{T_{i,1},T_{i,2},TF_{i,1},TF_{i,2}\right\}}_{i\in N})$, the fog node first computes:

$Q_{1}^{\prime }=T_{1}^{1/\beta }=K_{1}^{\alpha }$ and for each i∈N,

$$\begin{array}{cc} Q_{i,1}^{\prime }=T_{i,1}^{1/\beta }=K_{i,1}^{\alpha }, & QF_{i,1}^{\prime }=TF_{i,1}^{1/\beta }=F_{i,1}^{\alpha }\\ Q_{i,2}=T_{i,2}^{1/\beta }=K_{i,2}^{\alpha }, & QF_{i,2}=TF_{i,2}^{1/\beta }=F_{i,2}^{\alpha } \end{array}$$

Specially, for the same $i^{\prime }$,

$$\begin{array}{c} Q_{i^{\prime },1}^{\prime }=T_{i^{\prime },1}^{1/\beta }=K_{i^{\prime },1}^{\alpha H(w^{\prime })},QF_{i^{\prime },1}^{\prime }=TF_{i^{\prime },1}^{1/\beta }=F_{i^{\prime },1}^{\alpha H(w^{\prime })}\\ Q_{i^{\prime },2}=T_{i^{\prime },2}^{1/\beta }=K_{i^{\prime },2}^{\alpha H(w^{\prime })},QF_{i^{\prime },2}=TF_{i^{\prime },2}^{1/\beta }=F_{i^{\prime },2}^{\alpha H(w^{\prime })} \end{array}$$

Then, the fog node pre-decrypts the ciphertext $CT_{m}$ by the following calculations.

For each i∈I,

if $\underline{i}=i$ and i∈S
  $$\begin{array}{c} e\left(C_{i}^{*},Q_{i,1}^{\prime }\right)=e\left(g^{t_{i}s_{m}},g^{\left(r_{i}-x_{i}\right)\alpha /t_{i}}\right)=e{\left(g,g\right)}^{s_{m}\alpha \left(r_{i}-x_{i}\right)}\\ e\left(C_{i}^{*},Q_{i,2}\right)=e\left(g^{t_{i}s_{m}},g^{x_{i}\alpha /t_{i}}\right)=e{\left(g,g\right)}^{s_{m}\alpha x_{i}} \end{array}$$
if $\underline{i}=\neg i$ and i∉S
  $$\begin{array}{c} e\left(C_{i}^{*},Q_{i,1}^{\prime }\right)=e\left(g^{t_{i+n}s_{m}},g^{\left(r_{i}-x_{i}\right)\alpha /t_{i+n}}\right)=e{\left(g,g\right)}^{s_{m}\alpha \left(r_{i}-x_{i}\right)}\\ e\left(C_{i}^{*},Q_{i,2}\right)=e\left(g^{t_{i+n}s_{m}},g^{x_{i}\alpha /t_{i+n}}\right)=e{\left(g,g\right)}^{s_{m}\alpha x_{i}} \end{array}$$

for each i∉I,

$$\begin{array}{c} e\left(C_{i}^{*},QF_{i,1}^{\prime }\right)=e\left(g^{t_{i+2n}s_{m}},g^{\left(r_{i}-x_{i}\right)\alpha /t_{i+2n}}\right)=e{\left(g,g\right)}^{s_{m}\alpha \left(r_{i}-x_{i}\right)}\\ e\left(C_{i}^{*},QF_{i,2}\right)=e\left(g^{t_{i+2n}s_{m}},g^{x_{i}\alpha /t_{i+2n}}\right)=e{\left(g,g\right)}^{s_{m}\alpha x_{i}} \end{array}$$

for $i^{\prime }\in N$

$$\begin{array}{c} e\left(C_{i^{\prime }}^{*},Q_{i^{\prime },1}^{\prime }\right)=e\left(C_{i^{\prime }}^{*},QF_{i^{\prime },1}^{\prime }\right)=e{\left(g,g\right)}^{s_{m}\alpha \left(r_{i^{\prime }}-x_{i^{\prime }}\right)}\\ e\left(C_{i^{\prime }}^{*},Q_{i^{\prime },2}\right)=e\left(C_{i^{\prime }}^{*},QF_{i^{\prime },2}\right)=e{\left(g,g\right)}^{s_{m}\alpha x_{i^{\prime }}} \end{array}$$

aggregating the above values, the fog node gets

$$\begin{array}{ccc} e\left(\bar{C},Q_{i,1}^{{}^{\prime }}\right)\prod_{i\in N}e{\left(g,g\right)}^{s_{m}\alpha \left(r_{i}-x_{i}\right)}\cdot e{\left(g,g\right)}^{s_{m}\alpha x_{i}} & = & e\left(g^{s_{m}},g^{(y-r)\alpha }\right)\prod_{i\in N}e{\left(g,g\right)}^{s_{m}\alpha r_{i}}\\ & = & e{\left(g,g\right)}^{s_{m}y\alpha -s_{m}\alpha r}\cdot e{\left(g,g\right)}^{s_{m}\alpha r}\\ & = & e{\left(g,g\right)}^{s_{m}y\alpha }. \end{array}$$

Finally, the fog node sends the decryption result $CT^{*}=\left(C_{m},e{\left(g,g\right)}^{s_{m}y\alpha }\right)$ back to the user.

**Step 2: Data user decryption**

Once receives $CT^{*}$ from the fog node, the data user can get the message $m=C_{m}/\left(e{\left(g,g\right)}^{(s_{m}y\alpha )1/\alpha }\right)$.

### 3.4. User Revocation

To revoke a user from the current system, a data owner publishes the revoked user to user revocation list (URL) and asks the related fog nodes to delete this user from the related per-dataset ULs. After that, the related fog nodes would not answer any request from this user so that this user is revoked implicitly.

## 4. Security and Performance Analysis

### 4.1. Security Analysis

**Keyword privacy:** Duo to adoption of online/offline mechanism, the secure index generation of the proposed scheme is split into two phases. According to [23], the online/offline ABE scheme always extends some basic ABE scheme to support online/offline mechanism and its security can be reduced to the security of the basic ABE scheme. Our scheme can be considered as an extension of mediated ABKS (mABKS) constructed by combining mediated encryption mechanism with ABKS scheme in [16,34]. Therefore, the security of our scheme can be reduced to the security of mABKS scheme. Appendix A, we first prove the mABKS selectively secure against chosen keyword attack (CKA) under standard model and then the CKA security of the proposed scheme is proved. In addition, in the proposed scheme, although a data encryption module is also added into mABKS, it does not affect the security of the proposed scheme because the data ciphertext and index ciphertext have the same ciphertext structure and are randomized by different random fators.

**Data confidentiality:** According to above analysis, the data confidentiality under chosen-plaintext attack (CPA) is also ensured by the security of mABKS. The other threat to data confidentiality is collusion attacks from malicious users, in which multiple users collude to combine their keys to decrypt a ciphertext that none of them alone could. The collusion attack resistance is an important security property in ABE scheme. Our design adopts the same technique as in [26] to avoid collusion attack, where each secret key component related to some attribute *i* is independently randomized by a random factor $r_{i}\in Z_{p}$ and the key component $K_{1}=g^{y-r}$ (where $r=\sum r_{i})$ is used to bind all attribute secret key components of the same user together. In addition, in our scheme, the secret key of a user is split into two shares, under the assumption of no collusion between user and mediator, the user-side secret key components from different users will not match with mediator-side key share and thus is invalid.

**Trapdoor unlinkability:** In our scheme, data user randomizes each trapdoor using random numbers α. Therefore, cloud server and fog nodes will not be able to visually distinguish two or more trapdoors even containing the same keyword.

**Swapping attack resistance:** In our scheme, keyword and data are encrypted with the same access structure and key and the two ciphertexts also contain the same keyword element, thus the alteration of relationship between the two ciphertexts can be detected immediately.

### 4.2. Performance Analysis

We evaluate the performance of each phase of the proposed hybrid scheme in terms of asymptotic complexity and actual implementation efficiency. In particular, we make a comprehensive performance comparison between our proposed hybrid scheme with ABKS_UR scheme [16,34]. As done in [16,34], asymptotic complexity is measured in terms of the pairing operation *P*, the group exponentiation *E* and the group multiplication *M* in *G*, the group exponentiation $E_{T}$ and the group multiplication $M_{T}$ in $G_{T}$; the actual implementation efficiency is evaluated using the real-world Enron Email Dataset [35] containing about half million files from approximate 150 users and the elliptic curve of Type A with 160-bit group order and a level of 1024-bit DLOG security.

Our simulation includes two parts. First, to facilitate comparison, we conduct simulate experiments of system setup, user register, secure index generation, per-index search, user revocation for our scheme and ABKS_UR using the Charm Cryptography python library [36] on a MacBook Pro with an Intel Core i5 2 Duo 1.4 GHz and 4 GB RAM. Moreover, under the simulation environment, we also compare the implementation efficiencies of two steps in data encryption and decryption process of our scheme, respectively. Then, in order to show the feasibility of our scheme in fog environment, we implement the most complex user-side online computation task of our scheme, trapdoor generation computation on the user side, on an Android smartphone with 2.0 GHz ARM-based Nubia Z11 minis with 4 GB RAM running Android 6.1 OS using the JPBC library [37].

#### 4.2.1. The Efficiency of System Initialization

As shown in Table 2, our scheme has the same system setup cost as ABKS_UR and the main computation overhead includes 3*n* exponentiations in *G*, one exponentiation in $G_{T}$ and one pairing operation on the AA side, and is linear to the number of attributes in the system. Because the user secret key is split into two parts for implementing the mediator mechanism in our schemes, compared with ABKS_UR, additional 2*n* exponentiation operations in *G* are implemented to generate mediator-side share. In addition, when an entity wants to join the system as a mediator, he has to request AA for identity key and request data owner for dataset-mediator public key. The two key generations incur one exponentiation in $G_{T}$, respectively.

**Figure 2:**
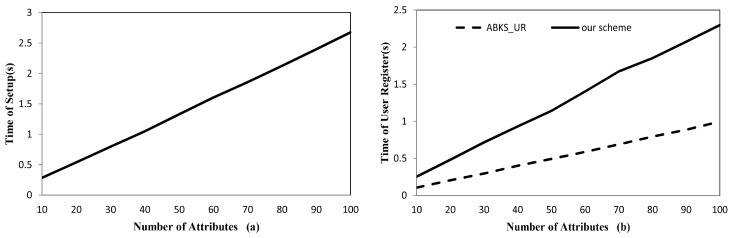
(**a**) Time for system setup. (**b**) Time for user register.

**Implementation.** The simulation results are shown in Figure 2. Figure 2a shows that our scheme and ABKS_UR have the same system setup cost, which is linear to the number of attributes in the system. Figure 2b shows that the user register time of our scheme is nearly twice as long as that of ABKS_UR due to key separation mechanism in our scheme. When the number of attributes in the system is 100, our scheme is about 1.2 s slower than ABKS_UR. However, all computation tasks in this phase are performed on AA side, where more computing power will greatly reduce the implementation time and the time gap will far less than 1 s. Therefore, the efficiency in this phase should be acceptable in practice.

#### 4.2.2. The Efficiency of Data Outsourcing

In this phase, the addition operation *A* in $Z_{p}$ is also considered as an indicator of asymptotic complexity. By using online/offline ABE technique, Table 3 shows that the offline computation of our scheme does the vast majority of the work to encrypt index and data file before knowing the data and access policy and the main online operation is addition in $Z_{p}$.

**Figure 3:**
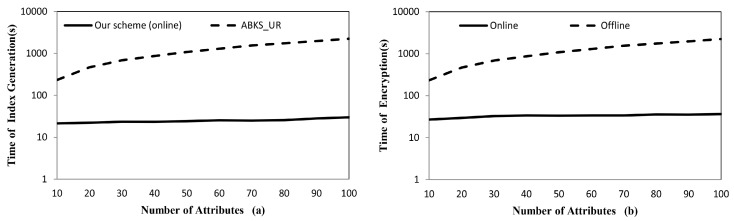
(**a**) Secure index generation time for 10,000 items. (**b**) Online and offline data encryption time for 10,000 items.

**Implementation.** In Figure 3, we give the implementation results of secure index generation and data encryption for 10,000 data items. Figure 3a shows that the time cost for online secure index generation on owner side in our scheme is far less than index generation cost in ABKS_UR. In Figure 3b, we compare online and offline data encryption cost and the result shows that the online computation only takes tiny share of the total data encryption time. Therefore, the online/offline mechanism indeed can remove the possible performance bottleneck on the resource constrained devices.

#### 4.2.3. The Efficiency of Data Search and Access

As shown in Table 4, in this phase, adoption of mediator mechanism incurs more trapdoor generation cost on user side in our scheme than ABKS_UR. At the same time, the mediator and online/offline mechanism also incur more computation overhead in search process of our scheme and the increased costs include *n* pairings, 2n exponentiations in *G* and one multiplication in $G_{T}$ on server side. In addition, in our scheme, the vast majority of data decryption task is outsourced to fog node and the end user only need to perform one exponentiation and one multiplication in $G_{T}$.

**Figure 4:**
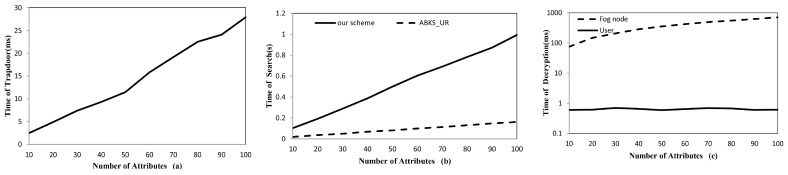
(**a**) User-side time for trapdoor generation on an Android smartphone. (**b**) Time for per-index search. (**c**) Time for pre-decryption on fog node side and decryption on user side.

**Implementation.** In order to demonstrate the feasibility of our scheme in fog environment, we separately program the trapdoor generation process on the user side on an Android smartphone using the JPBC library. Since there is no pairing operation in trapdoor generation process, Figure 4a shows that the time cost is less than 30 ms when the number of attributes in the system is 100, thus the computation on the user side is still very efficient. For per-index search, Figure 4b shows that our scheme is about 0.8 s slower than ABKS_UR When the number of attributes in the system is 100. Since search process is implemented on server side with powerful computing capacity, the time gap will far less than the experimental result and should be totally acceptable in practice. From Figure 4c, we can find that the data decryption task that data user undertakes is far less than fog node, thus the data users will be relaxed even if they have limited resources.

#### 4.2.4. User Revocation

In our scheme, when a user revocation occurs, a data owner publishes the revoked user to user revocation list (URL) and then the related fog nodes delete this user from the corresponding per-dataset ULs, thus, the user revocation cost is independent of the number of ciphertexts and users in system. However, in ABKS_UR, a user revocation will need to update the keys of non-revoked users who hold the revoked attributes and ciphertexts which contain the revoked attributes, thus the revocation cost is linear to the number of users and ciphertexts in the system. Let $N_{u}$ be the number of users and $N_{f}$ be the total number of secure ciphertexts in the system and when a user revocation occurs, let $n_{u}$ denote the number of non-revoked users who hold the revoked attributes and $n_{f}$ be the number of ciphertexts which contain the revoked attributes, the cost of a user revocation in ABKS_UR is $n_{u}x\left(M\;+\;E\right)+n_{f}yE\;+\;n_{u}zE,1\leq x,y,z\leq n,1\leq n_{f}\leq N_{f},1\leq n_{u}\leq N_{u}$. Therefore, with increase of the number of users/ciphertexts in system, the user revocation cost will rapidly rise.

In summary, our scheme greatly reduces the workload on the data owner over online time and on the data user for data decryption. The data owner only performs one group multiplication, one group exponentiation, multiple additions in $Z_{p}$ for secure index generation and two group multiplications, one group exponentiation, multiple additions in $Z_{p}$ for data encryption in the online phase, meanwhile, the data user only performs one group exponentiation and one group multiplication for data decryption. Apparently, these properties will bring great benefits for resource restrained end users. Although the computation costs for key generation, trapdoor generation and per-index search increase due to the introduction of mediated mechanism and online/offline ABE technique, these computation costs are only linearly related with the number of attributes rather than the number of users and data files in the system. Generally speaking, the number of attributes in a system is often far less than the number of the users and data files, thus, the overall efficiency of our scheme is totally acceptable in practice.

## 5. Conclusions

In this paper, we provide a hybrid solution for fine-grained owner-enforced search and access authorization spanning user-fog-cloud and meeting the resource constraint of end devices. In the proposed scheme, the index encryption and data encryption components are bound together by sharing the same key and keyword element, thus the cost of key management greatly decreases and the access efficiency is significantly improved. In addition, in our scheme, fog nodes undertake instantaneous user revocation and pre-decryption, meanwhile resource constrained end devices can easily complete encryption and decryption task online. Therefore, our scheme is perfectly suitable for IoT applications in a fog computing environment. To the best of our knowledge, our scheme is the first hybrid scheme designed for a fog computing environment. Although the proposed scheme is constructed for single-keyword search request, it is easy to provide conjunctive keyword search functionality as noted in [16,34]. The limitation of the proposed schemes is that access policy consists of simple AND gates and the system architecture is centralized (single AA). In the future, the scheme with more expressive access policy and more flexible architecture, such as hierarchical or multi-authority architecture, may be needed.

## Figures and Tables

**Table 1:** Comparison of related works.

References	Single User	Multiple Users	Index Encryption	Data Encryption	Instantaneous User
					Revocation
[11]	√			√	
[13]	√		√		
[17]		√	√		
[15,33]		√	√		√
[16,34]		√	√		
[19]		√	√	√	

**Table 2:** Asymptotic Complexity Comparison of System Initialization.

Schemes	System Setup	User Register	Mediator Register
our scheme	$P\;+\;3nE\;+\;E_{T}$	$\left(4n\;+\;1\right)E\;+\;2E_{T}$	$2E_{T}$
ABKS_UR	$P\;+\;3nE\;+\;E_{T}$	$\left(2n\;+\;1\right)E\;+\;2E_{T}$	∖

**Table 3:** Asymptotic Complexity Comparison of Data Outsourcing Process.

Schemes	Secure Index Generation		Ciphertext Generation	
	Online	Offline	Online	Offline
our scheme	M+E+nA	$\left(n+1\right)E\;+E_{T}$	$M\;+M_{T}\;+E\;+nA$	$\left(n+1\right)E\;+E_{T}$
ABKS_UR	$\left(n+1\right)E\;+\;E_{T}$		∖	

**Table 4:** Asymptotic Complexity Comparison of Data Search and Access Process.

Schemes	Trapdoor Generation		Per-Index Search	Data Decryption	
	User	Fog Node		User	Fog Node
our scheme	(4n+1)E	$\left(4n\;+\;1\right)E\;+\;E_{T}$	(2n+1)P+2nE+	$E_{T}\;+\;M_{T}$	(2n+1)P+
			$\left(n\;+\;3\right)M_{T}\;+\;E_{T}$	$\;\;\left(2n\;+\;1\right)E\;+\;\left(n\;+\;1\right)M_{T}\;$	
ABKS_UR	(2n+1)E	∖	$\left(n\;+\;1\right)P\;+\;\left(n\;+\;2\right)M_{T}\;+\;E_{T}$	∖	∖

## Appendix A. Security Proof

We will prove our scheme under the DBDH (Decisional Bilinear Diffie-Hellman) assumption.

**DBDH** **Assumption:** *Let* $a,b,c,z\in Z_{p}$ *be chosen at random and g be a generator of G. The DBDH assumption is that no probabilistic polynomial-time adversary* B *can distinguish the tuple* $\left(A=g^{a},B=g^{b},C=g^{c},e{\left(g,g\right)}^{abc}\right)$ *from the tuple* $\left(A=g^{a},B=g^{b},C=g^{c},e{\left(g,g\right)}^{z}\right)$ *with non-negligible advantage. The advantage of* B *is defined as* $|Pr\left[B\left(A,B,C,e{\left(g,g\right)}^{abc}\right)=0\right]-Pr\left[B\left(A,B,C,e{\left(g,g\right)}^{z}\right)=0\right]|$*, where the probability is taken over the random choice of the generator g, the random choice of* a,b,c,z *in* $Z_{p}$ *and the random bits consumed by* B*.*

Before proving our scheme, we need to prove a simpler version of our scheme, which extends ABKS scheme in [16,34] to support mediator mechanism, namely mediated ABKS (mABKS). Note that we does not consider the user revocation here.

### Appendix A.1. Security of mABKS

#### Appendix A.1.1. Scheme Description

Compared to the proposed shceme in Section 3, the online/offline encryption mechanism and data encryption are not considered in mABKS, thus, in search phase, there is no data decryption. In the following, we give a brief description for mABKS.

- **System Initialization.** This phase in mABKS is the exact same as that in the proposed scheme in Section 3.
- **Secure Index Generation.** Data owner generates the secure index of some keyword for his each outsourced file. Like the proposed scheme, mABKS scheme also adopts a two-layer authorization structure for accelerating search. For a dataset, the data owner first selects a secret value $s\in Z_{p}$ randomly and computes $D_{0}=Y^{s}$ and $\bar{D}=g^{s}$. Let *w* denote the keyword from a file in the dataset and $GT=\wedge_{i\in I}\underline{i}$ be a designated access structure for the file, for each i∈N, the data owner computes
  $$D_{i}=\left\{\begin{array}{cc} A_{i}^{s} & i\in I,\;\underline{i}=i\\ A_{i+n}^{s} & i\in I,\;\underline{i}=\neg i\\ A_{i+2n}^{s} & i\in N\setminus I \end{array}\right.$$
  The keyword *w* is encrypted in the following way. For some attribute $i^{\prime }\in N$, the data owner calculates $D_{i^{\prime }}=A_{i^{\prime }}^{s/H(w)}$. Finally, the secure index $D=(GT,D_{0},\bar{D},{\left\{D_{i}\right\}}_{i\in N})$ is sent to cloud server along with the encrypted file (the file encryption isn’t considered in this scheme).
- **Trapdoor Generation.** Trapdoor generation process in mABKS is exactly the same as that in the proposed scheme in Section 3.
- **Search.** Substituting ${\left\{D_{i}\right\}}_{i\in N}$ for ${\left\{D_{i}^{*}\right\}}_{i\in N}$ in Section 3.3.2, search process in mABKS can be implemented using Step 2 and Step 3 of the search process in the proposed scheme.

#### Appendix A.1.2. Security Definition for mABKS

**Definition** **1.** A mABKS scheme is said to be selectively secure against chosen-keyward attack (CKA) if no probabilistic polynomial-time adversary has non-negligible advantage in the following Game-1.

**Game-1**

**Init.** The adversary A submits a challenge access structure $GT^{*}$ to the challenger B.

**Setup.** B runs system setup operation to obtain the public parameters PP and master key MSK for the system. Then B sends PP to A.

**Phase 1.** By transmitting any keyword *w* and any attribute set *S* (*S* cannot satisfy the challenge access structure $GT^{*}$), A is allowed to request repeatedly to any of the following queries:

- ${\mathrm{TrapGen}}^{1}\left(S\right)$: A asks for a mediator share of trapdoor for a chosen attribute set *S* and receives $T_{med}$.
- ${\mathrm{TrapGen}}^{2}\left(S,w\right)$: A asks for a user share of trapdoor for a chosen attribute set *S* and keyword *w*, then receives $T_{u}$.
- ${\mathrm{TrapGen}}^{3}\left(S,w\right)$: A asks for a complete value of trapdoor for a chosen attribute set *S* and keyword *w*, then receives the whole trapdoor *T*.

**Challenge.** A submits two keywords $w_{0}$ and $w_{1}$ with the same length. Then B defines a coin σ∈{0,1} and encrypts $w_{\sigma }$ with the challenge structure $GT^{*}$. Finally, the ciphertext is sent back to A.

**Phase 2.** Phase 1 is repeated, but the keywords $w_{0}$ and $w_{1}$ in challenge phase should not be requested.

**Guess.** A outputs the guess $\sigma^{\prime }$.

#### Appendix A.1.3. Security Proof for mABKS

**Theorem** **1.** *We can construct a simulator to solve the DBDH problem with non-negligible advantage* ϵ/2 *if there is a probabilistic polynomial-time adversary wins Game-1 with non-negligible advantage ϵ. That is to say, the DBDH security assumption ensures the security of mABKS.*

**Proof.** Assume that the system security parameter is λ and the universe attribute set is *N*. The DBDH challenger first chooses $a,b,c,z\in Z_{p}$ randomly and a fair coin ν∈{0,1}. It sets $Z=e{\left(g,g\right)}^{abc}$ if ν=0 and $Z=e{\left(g,g\right)}^{z}$ otherwise.Then the simulator B gets a tuple $\left(A,B,C,Z\right)=\left(g^{a},g^{b},g^{c},Z\right)$ from the challenger and outputs ν. Now the simulator B plays a challenger in the following game. **Init.** The adversary A first submits the access structure $GT^{*}=\wedge_{i\in I}\underline{i}$ which he wants to challenge to the simulator B. **Setup.** B generates the public parameters of the system. Let $Y=e\left(A,B\right)=e{\left(g,g\right)}^{ab}$, which implicitly sets y=ab. It chooses θ=u,t=γ from $Z_{p}$, and defines $PP_{UID}=e{\left(A,B\right)}^{\theta },PP_{med}=e{\left(A,B\right)}^{t}$. For each i∈N, B chooses $\alpha_{i},\beta_{i},\gamma_{i}\in Z_{p}$ and outputs the following parameters.

$$\begin{array}{c} \mathrm{For}\;i\;\in I\;\mathrm{and}\;\underline{i}=i,A_{i}=g^{\alpha_{i}},A_{i+n}=B^{\beta_{i}},A_{i+2n}=B^{\gamma_{i}}\\ \mathrm{For}\;i\;\in I\;\mathrm{and}\;\underline{i}=\neg i,A_{i}=B^{\alpha_{i}},A_{i+n}=g^{\beta_{i}},A_{i+2n}=B^{\gamma_{i}}\\ \mathrm{For}\;i\;\notin I,A_{i}=B^{\alpha_{i}},A_{i+n}=B^{\beta_{i}},A_{i+2n}=g^{\gamma_{i}} \end{array}$$

Finally, B publishes the above parameters. **Phase 1.** A can ask B for a trapdoor for any keyword *w* and attribute set *S* which should not satisfy the challenge access structure $GT^{*}$. B uses the collision resistant hash function to output H(w). It is clear that there is a witness attribute j∈I such that either j∈S and $\underline{j}=\neg j$, or j∉S and $\underline{j}=j$ because *S* dose not satisfy $GT^{*}$. Without loss of generality, we assume j∉S and $\underline{j}=j$.

- $B^{\prime }s\;\mathrm{response}\;\mathrm{to}\;\mathrm{the}\;\mathrm{request}\;\mathrm{for}\;\mathrm{the}\;\mathrm{mediator}\;\mathrm{share}\;\mathrm{of}\;a\;\mathrm{trapdoor}:$ For all i∈N, B randomly chooses $r_{i}^{\prime },x_{i}^{\prime }\in Z_{p}$ and sets $x_{i}=bx_{i}^{\prime }$, $r_{j}=ab+br_{j}^{\prime }$ and $r_{i}=br_{i}^{\prime }$ if i≠j. Then, B computes $r=\prod_{i=1}^{n}r_{i}=ab+\prod_{i=1}^{n}br_{i}^{\prime }$ and chooses f=β from $Z_{p}$ as a random secret value of the mediator. Consequently, B can compute $Q_{1}=K_{1}^{\beta }=B^{-f\sum_{i=1}^{n}r_{i}^{\prime }}=g^{-f\sum_{i=1}^{n}br_{i}^{\prime }}$, and for the witness attribute j∈S such that j∉S and $\underline{j}=j$, compute $Q_{j,1}=A^{\frac{f}{\beta_{j}}}\cdot g^{\frac{r_{j}^{\prime }-x_{j}^{\prime }}{\beta_{j}}f}=g^{\frac{ab+br_{j}^{\prime }-bx_{j}^{\prime }}{b\beta_{j}}f}=g^{\frac{r_{j}-x_{j}}{b\beta_{j}}f}$ and $QF_{j,1}=A^{\frac{f}{\gamma_{j}}}\cdot g^{\frac{r_{j}^{\prime }-x_{j}^{\prime }}{\gamma_{j}}f}=g^{\frac{ab+br_{j}^{\prime }-bx_{j}^{\prime }}{b\gamma_{j}}f}=g^{\frac{r_{j}-x_{j}}{b\gamma_{j}}f}$.
  For i≠j, B has: 1) i∈S. if $i\in I\wedge \underline{i}=i$, $Q_{i,1}=B^{\frac{r_{i}^{\prime }-x_{i}^{\prime }}{\alpha_{i}}f}=g^{\frac{br_{i}^{\prime }-bx_{i}^{\prime }}{\alpha_{i}}f}=g^{\frac{r_{i}-x_{i}}{\alpha_{i}}f}$ and if $(i\in I\wedge \underline{i}=\neg i)\vee i\notin I$, $Q_{i,1}=g^{\frac{r_{i}^{\prime }-x_{i}^{\prime }}{\alpha_{i}}f}=g^{\frac{r_{i}-x_{i}}{b\alpha_{i}}f}$.
  2) i∉S. if $\left(i\in I\wedge \underline{i}=\neg i\right)$, $Q_{i,1}=B^{\frac{r_{i}^{\prime }-x_{i}^{\prime }}{\beta_{i}}f}=g^{\frac{br_{i}^{\prime }-bx_{i}^{\prime }}{\beta_{i}}f}=g^{\frac{r_{i}-x_{i}}{\beta_{i}}f}$ and if $(i\in I\wedge \underline{i}=i)\vee i\notin I$, $Q_{i,1}=g^{\frac{r_{i}^{\prime }-x_{i}^{\prime }}{\beta_{i}}f}=g^{\frac{r_{i}-x_{i}}{b\beta_{i}}f}$.
  Meanwhile, B also computes $QF_{i,1}$ components similarly for i≠j, if $i\in I,QF_{i,1}=g^{\frac{r_{i}^{\prime }-x_{i}^{\prime }}{\gamma_{i}}f}=g^{\frac{r_{i}-x_{i}}{b\gamma_{i}}f}$ and if i∉I, $QF_{i,1}=B^{\frac{r_{i}^{\prime }-x_{i}^{\prime }}{\gamma_{i}}f}=g^{\frac{r_{i}-x_{i}}{\gamma_{i}}f}$. Finally, $B^{\prime }s$ response is $T_{sem}=\left(Q_{1},{\left\{Q_{i,1},QF_{i,1}\right\}}_{i\in N}\right)$
- $B^{\prime }s\;\mathrm{response}\;\mathrm{to}\;\mathrm{the}\;\mathrm{request}\;\mathrm{for}\;\mathrm{the}\;\mathrm{user}\;\mathrm{share}\;\mathrm{of}\;a\;\mathrm{trapdoor}:$B chooses α=h from $Z_{p}$ as a random secret value of the user and gets $Q_{0}=u+\alpha =\theta +h$, $T_{1}=Q_{1}^{h}$, and for all i∈N except for $i^{\prime }$, $T_{i,1}=Q_{i,1}^{h},TF_{i,1}=QF_{i,1}^{h}$. For the witness attribute j∈I∖S, and $\underline{j}=j$, B denotes $Q_{j,2}=g^{\frac{x_{j}^{\prime }}{\beta_{j}}h}=g^{\frac{bx_{j}^{\prime }}{b\beta_{j}}h}=g^{\frac{x_{j}}{b\beta_{j}}h}$ and $QF_{j,2}=g^{\frac{x_{j}^{\prime }}{\gamma_{j}}h}=g^{\frac{x_{j}}{b\gamma_{j}}h}$.
  Except for $i^{\prime }$, for i≠j, the $Q_{i,2}$ components can be computed as follows:
  (1) i∈S. If $i\in I\wedge \underline{i}=i$ $Q_{i,2}=B^{\frac{x_{i}^{\prime }}{\alpha_{i}}h}=g^{\frac{x_{i}}{\alpha_{i}}h}$; if $(i\in I\wedge \underline{i}=i)\vee i\notin I$ $Q_{i,2}=g^{\frac{x_{i}^{\prime }}{\alpha_{i}}h}=g^{\frac{x_{i}}{b\alpha_{i}}h}$.
  (2) i∉S. If $i\in I\wedge \underline{i}=\neg i$, $Q_{i,2}=B^{\frac{x_{i}^{\prime }}{\beta_{i}}h}=g^{\frac{bx_{i}^{\prime }}{\beta_{i}}h}=g^{\frac{x_{i}}{\beta_{i}}h}$; if $(i\in I\wedge \underline{i}=i)\vee i\notin I$, $Q_{i,2}=g^{\frac{x_{i}^{\prime }}{\beta_{i}}h}=g^{\frac{x_{i}}{b\beta_{i}}h}$.
  Similarly, except for $i^{\prime }$, for j≠j, B also computes $QF_{i,2}=g^{\frac{x_{i}^{\prime }}{\gamma_{i}}h}=g^{\frac{x_{i}}{b\gamma_{i}}h}$ if i∈I and $QF_{i,2}=B^{\frac{x_{i}^{\prime }}{\gamma_{i}}h}=g^{\frac{x_{i}}{\gamma_{i}}h}$ if i∉I. Without loss of generality, consider $i^{\prime }\in S\cap I$ and $\underline{i^{\prime }}=i^{\prime }$. Thus, B sets $T_{i^{\prime },1}=Q_{i^{\prime },1}^{hH(w)},TF_{i^{\prime },1}=QF_{i^{\prime },1}^{hH(w)},Q_{i^{\prime },2}=B^{\frac{bx_{i^{\prime }}^{\prime }}{\alpha_{i^{\prime }}}hH\left(w\right)}=g^{\frac{x_{i^{\prime }}}{\alpha_{i^{\prime }}}hH\left(w\right)},QF_{i^{\prime },2}=g^{\frac{x_{i^{\prime }}^{\prime }}{\gamma_{i^{\prime }}}hH\left(w\right)}=g^{\frac{x_{i^{\prime }}}{b\gamma_{i^{\prime }}}hH\left(w\right)}$.
- $B^{\prime }s\;\mathrm{response}\;\mathrm{to}\;\mathrm{the}\;\mathrm{request}\;\mathrm{for}\;a\;\mathrm{complete}\;\mathrm{trapdoor}:$B sets γ=t and computes $T_{0}=fQ_{0}+t,D_{med}^{set}=Z^{-t},T_{UID}^{set}=Z^{-f\theta }$. For all i∈N, B computes $T_{i,2}=Q_{i,2}^{f},TF_{i,2}=QF_{i,2}^{f}$. Finally, $B^{\prime }s$ response is $T=(D_{med}^{set},T_{UID}^{set},T_{0},T_{1},\{T_{i,1},T_{i,2},TF_{i,1},$ $TF_{i,2}{\}}_{i\in N})$.

**Challenge.** Once receives two challenge keywords $w_{0},w_{1}$ with the same length from A, B defines a fair coin σ∈{0,1} and then encrypts $w_{\sigma }$ with the challenge policy $GT^{*}$ as follows. B sets $D_{0}=Z,\bar{D}=C$ and then except for $i^{\prime }$, for i∈I, computes the $D_{i}$ components as follows. $D_{i}=C^{\alpha_{i}}\;if\;\underline{i}=i$, $D_{i}=C^{\beta_{i}}\;if\;\underline{i}=\neg i$, and for $i\notin I,D_{i}=C^{\gamma_{i}}$. Without loss of generality, we assume $i^{\prime }\in I\;and\;\underline{i^{\prime }}=i^{\prime }$ and compute $D_{i^{\prime }}=C^{\alpha_{i^{\prime }}/H\left(w_{\sigma }\right)}$. Consequently, B sends $CT^{*}=\left(D_{0},\bar{D},{\left\{D_{i}\right\}}_{i\in N}\right)$ to A. **Phase 2.** B proceeds as phase 1, but keywords should not be same as ones in challenge phase. **Guess.** A outputs a guess $\sigma^{\prime }$. If $\sigma^{\prime }=\sigma$ the simulator will output $\nu^{\prime }=0$ to indicate that it was given a valid DBDH-tuple otherwise it will output $\nu^{\prime }=1$ to indicate it was given a random 4-tuple. In the case where ν=1, A gains no information about σ. Therefore, we have $Pr\left[\sigma \neq \sigma^{\prime }|\nu =1\right]=\frac{1}{2}$. As B guesses $\nu^{\prime }=1$ when $\sigma \neq \sigma^{\prime }$, we have $Pr\left[\nu =\nu^{\prime }|\nu =1\right]=Pr\left[\nu^{\prime }=1|\nu =1\right]=\frac{1}{2}$. In the case where $\nu^{\prime }=0$, A is given a valid encryption of $w_{\sigma }$ and it can win the game with non-negligible advantage ϵ and thus we have $Pr\left[\sigma =\sigma^{\prime }|\nu =0\right]=\frac{1}{2}+\epsilon$. As B guesses $\nu =\nu^{\prime }$ when $\sigma =\sigma^{\prime }$, we have $Pr\left[\nu^{\prime }=\nu |\nu =0\right]=Pr\left[\nu^{\prime }=0|\nu =0\right]=\frac{1}{2}+\epsilon$. Hence, we can conclude that the advantage of B in the DBDH game is $Pr\left[\nu^{\prime }=\nu \right]-\frac{1}{2}=Pr\left[\nu =\nu^{\prime }|\nu =1\right]Pr\left[\nu =1\right]+Pr\left[\nu^{\prime }=\nu |\nu =0\right]Pr\left[\nu =0\right]-\frac{1}{2}=\frac{\epsilon }{2}$. ☐

### Appendix A.2. Security of the Proposed Scheme

#### Appendix A.2.1. Security Definition for the Proposed Scheme

**Definition** **2.** The proposed scheme can be said to be selectively secure against CKA and CPA if no PPT adversary has non-negligible advantage in the following Game-2.

**Game-2**

**Init.** The adversary A submits a challenge access structure $GT^{*}$ to the challenger B.

**Setup.** It is the same as Setup in Game-1.

**Phase 1.** It is the same as Phase 1 in Game-1.

**Challenge.** A submits two distinct message-keyword pairs $\left(w_{0},m_{0}\right)$ and $\left(w_{1},m_{1}\right)$, where $m_{0}$ and $m_{1}$ are the same-length messages and $w_{0}$ and $w_{1}$ are the same-length keywords. Then B defines a coin σ∈{0,1} and generates ciphertext $CT^{*}$ using the approach in Section 3.3. Finally, the ciphertext $CT^{*}$ is sent back to the adversary A.

**Phase 2.** Phase 1 is repeated, but the keywords should not be same as ones in challenge phase.

**Guess.** A outputs the guess $\sigma^{\prime }$.

**Theorem** **2.** We can construct a simulator to win Game-1 with non-negligible advantage ϵ if there is a probabilistic polynomial-time adversary wins Game-2 with non-negligible advantage ϵ. That is to say, the security of the proposed scheme can be reduced to the security of mABKS.

#### Appendix A.2.2. Security Proof for the Proposed Scheme

**Proof.** In order to proof Theorem 2, we will show that any PPT adversary A with non-negligible advantage in Game-2 can be used to break the selectively secure against CKA of mABKS scheme with a PPT simulator B. The simulator acts as a challenger and interacts with A in Game-2 with security parameter λ and the universe attribute set *N*. **Init.** Initially, B receives the access structure $GT^{*}$ from A and passes it to the mABKS challenger. **Setup.** Then B receives the public parameters PP from the mABKS challenger and passes them to A. **Phase 1.** The trapdoors are the same in both schemes, so any trapdoor generation request from A is passed by B to the mABKS challenger and B returns directly the trapdoors to A. **Challenge.** A submits two distinct message-keyword pairs $\left(m_{0},w_{0}\right),\left(m_{1},w_{1}\right)$, where $m_{0}$ and $m_{1}$ are the same-length messages and $w_{0}$ and $w_{1}$ are the same-length keywords, to B. Then B gives two keywords $w_{0},w_{1}$ to the mABKS challenger and receives back the mABKS challenge ciphertext $D^{*}=\left(GT^{*},D_{0},\bar{D},{\left\{D_{i}\right\}}_{i\in N}\right)$, where $D_{0}=e{\left(g,g\right)}^{ys},\bar{D}=g^{s}$. For each i∈N, $D_{i}$ is shown in Equation (1).

$$D_{i}=\left\{\begin{array}{cc} A_{i}^{s} & i\in I,\underline{i}=i\\ A_{i+n}^{s} & i\in I,\underline{i}=\neg i\;(1),\\ A_{i+2n}^{s} & i\in N/I \end{array}\right.\;\begin{array}{c} D_{i,1}=D_{i}\cdot A_{i}^{-z_{i1}},D_{i,2}=z_{i1}\\ C_{i,1}=D_{i}^{s^{\prime }}\cdot A_{i}^{-z_{i2}},C_{i,2}=z_{i2} \end{array}(2)$$

Specially, for $i^{\prime }\in N,D_{i^{\prime }}=A_{i^{\prime }}^{s/H(w_{\sigma })}$. Next, B selects random values $s^{\prime }$ and for each i∈N, $z_{i1},z_{i2}$ from $Z_{p}$ and computes $D_{i,1},C_{i,1},D_{i,2},C_{i,2}$ as shown in Equation (2). For special $i^{\prime }\in N$, B guesses a $\sigma_{B}$ and sets $D_{i^{\prime },2}=z_{i^{\prime }1}/H\left(w_{\sigma_{B}}\right),C_{i^{\prime },2}=z_{i^{\prime }2}/H\left(w_{\sigma_{B}}\right)$. He also has $C_{m_{\sigma_{B}}}=m_{\sigma_{B}}D_{0}^{s^{\prime }},\bar{C}={\bar{D}}^{s^{\prime }}$. Then, he sets $\tilde{CT_{w}^{*}}=\left(GT^{*},D_{0},\bar{D},{\left\{D_{i,1},D_{i,2}\right\}}_{i\in N}\right)$ and $\tilde{CT_{m}^{*}}=\left(GT^{*},C_{m_{\sigma_{B}}},\bar{C},{\left\{C_{i,1},C_{i,2}\right\}}_{i\in N}\right)$. The ciphertext is $\tilde{CT^{*}}=\left(\tilde{CT_{m}^{*}},\tilde{CT_{w}^{*}}\right)$. Finally, B sends $\tilde{CT^{*}}$ to A. **Phase 2.** B proceeds as Phase 1 while the requested keywords should not be challenged in the Challenge phase. **Guess.** A outputs the guess $\sigma_{A}$. If $\sigma_{A}=\sigma_{B}$, then B outputs $\sigma_{B}$. If $\sigma_{A}\neq \sigma_{B}$, then B outputs $1-\sigma_{B}$. Thus, if A has advantage ϵ in Game-2, B breaks the mABKS scheme with the same probability. ☐
